# National human genome projects: an update and an agenda

**DOI:** 10.4178/epih.e2017045

**Published:** 2017-10-16

**Authors:** Joon Yong An

**Affiliations:** Department of Psychiatry, Weill Institute for Neurosciences, University of California, San Francisco, CA, USA

**Keywords:** Genomics, Human genome project, Next generation sequencing, Whole genome sequencing, Genetics, Microarray

## Abstract

Population genetic and human genetic studies are being accelerated with genome technology and data sharing. Accordingly, in the past 10 years, several countries have initiated genetic research using genome technology and identified the genetic architecture of the ethnic groups living in the corresponding country or suggested the genetic foundation of a social phenomenon. Genetic research has been conducted from epidemiological studies that previously described the health or disease conditions in defined population. This perspective summarizes national genome projects conducted in the past 10 years and introduces case studies to utilize genomic data in genetic research.

## INTRODUCTION

Over the century, genetics studies have accounted for phenomena occurring in human society with genetic factors. In particular, with the development of genome sequencing technology, microarray and next generation sequencing, active research in the last decade has been conducted to identify genetic causes of human disorders and traits. Furthermore, international genomics consortiums have initiated and made an effort to make data sharing and research participations in the field. For example, the 1000 Genomes Project provided genomic information of 5 major human population groups and 26 specific population groups [1]. This consortium has released low coverage (2-4 folds depth) whole genome sequencing (WGS) data of individual population groups in a stepwise fashion from 2009 through 2015. In doing so, researchers have been able to study genetic variants specific to certain ancestry (e.g., fixed allele), or the distribution of rare variants present in less than 1% of the particular population. The WGS data of the 1000 Genome Project can be used as a reference set to impute genotypes of the population presumably with similar genetic background. Currently, several countries have conducted the genome projects on the genetic architecture of their population groups using WGS analyses.

National genome research provides an opportunity to describe the genetic background of inhabitants within the country. Investigation of traits or disorders can facilitate understanding the genetic distribution of the population within the country. Therefore, genomic data can be a useful resource for the health care and support, particularly those who are with a high-risk variant—patients with an extremely rare condition, etc.—can be received the primary care based on the research outcome. Thus, a national genome project is a tool to describe the population groups constituting a country but also understand confronting social phenomena in the community.

This perspective will introduce currently ongoing national genome projects. In particular, this will focus on national genome projects based on the next generation sequencing approach (whole exome sequencing [WES] and WGS), with particular concerns on the project rationales, data collection method, and plans to utilize the data. In addition, genetic studies based on the data generated from a national genome project will be discussed as an example. This perspective will suggest a general guideline of genome analysis and data utilization, which may be helpful in future genomic research based on WGS or WES in Korea, and will present collaborative efforts for national genome projects.

## RATIONALES FOR NATIONAL GENOME PROJECTS

### Genetic background of the people living in a country

Genomic research investigates the genetic background of inhabitants or facilitates understanding the consequences of a social phenomena within a population based on their genetic composition. Several European countries, including Iceland and the Netherlands, have analyzed genomic information to study people who have long resided in their area. National genome project in Iceland took advantage of family history and population characteristics of geographical isolation and have brought fundamental questions regarding human genetics and population genetics. On the other hand, the Netherlands national genome project categorized their population into sub-groups from 11 regions based on the history of city-states maintained in that country [2]. Finnish and Swedish national genome projects have identified heterogeneous genetic architectures in the inhabitants of their countries from historical events that influenced migration and fitness [3,4]. In the United Kingdom (UK), a national genome project focused on Pakistani immigrants from a multi-ethnic longitudinal cohort study, Born in Bradford, and given the assumption that immigrants might be genetically close, have performed WES analysis of 3,222 immigrants from the cohort to investigate whether homozygous variants attribute to particular trait or disease association [5].

### Genomic research to study disorders

A national genome project aims to establish clinical and genetic indices for diseases frequently reported in a certain population and to identify their risk factors. A variant occurring with a very low frequency in human population is population-specific, and might cause a disorder particularly observed in a certain population or to the inhabitants in a certain area [6]. However, it is necessary to consider whether genomic research of population provides rationale for investigation of particular disorders. This is because there is a wide range of genetic variants associated with or cause disorders. For example, common variants from genomewide association study and de novo variants from pedigree-based WES analysis have long been studied, and their genetic architecture and effect size well characterized by large-scale cohort studies. A rare variant observed a certain population could be genetic risk underlying population-specific traits or diseases. However, a large sample size is required for robust estimation for the effect size of a rare variant [7].

Therefore, a national genome project for clinical application should be sufficiently supported by a consideration of the genetic characteristics of the inhabitants, a link to epidemiological cohort research, or a genetic hypothesis. For instance, the Iceland national genome project investigated whether a protein-truncating variant affect the health condition as expected, considering that the population group is genetically homogeneous [8]. From WGS data of approximately 2,600 individuals, they made imputation the genotype of 100,000 individuals from the previous collection. They have collated rich information on family history, phenotype data, and mortality rate to examine which type of variants affect disorder, how low the frequency contribute to variant penetrance, and whether a homozygous variant—the type of variant representing geographical closeness and genetic similarity—actually attribute to a large effect size in the disorder. The success of the research was not attributable simply to using high-quality, recent WGS data. This is because the researchers have actively utilized previous genetic studies (i.e., genotype collection and genetic similarity estimation), and achieved the project with a hypothesis and appropriate experimental design in the context of their background (Table 1).

## NATIONAL GENOME PROJECTS: CASE STUDIES

### Iceland

**Figure 1.**
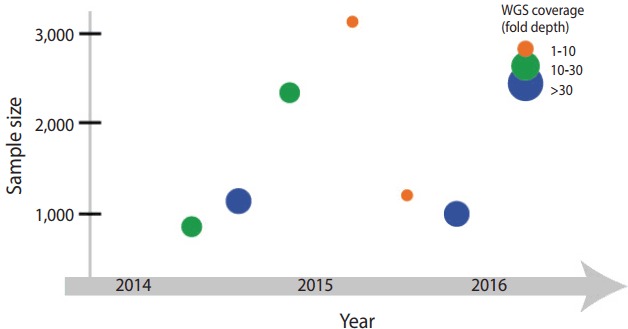
Progress in the national genome projects. Generation of whole genomic sequencing (WGS) data. The scatter plot illustrates the sample size (x-axis) and the year of completion (y-axis) for each project. The circle size is proportional to the coverage of WGS data.

Iceland is one of leading countries in national-level genome research, but also has contributed greatly to human genetics. Around 1996, before the draft of the Human Genome Project, an Icelandic venture company, deCODE genetics (https://www.decode.com), announced a plan to perform genome analysis on an entire population. Their plan seems solid as a low rate of genetic admixture due to geographical isolation that has been descent from about 20,000 emigrants from the northern Europe in the 9th century. It allowed an appropriate experimental design to study a founder effect derived from a small population. In addition, familial medical conditions and pedigree information have been recorded in detailed for many generations since the settlement. Taking advantage of the availability of rich data regarding family history and genetic homogeneity of the Icelandic population, the Icelandic national genome project has determined genetic factors underlying disorders or traits. With a whole genome data (Figure 1), they tested various theories and hypotheses of population genetics, such as paternal age effect on de novo variant, gene conversion error, and recessive model in health condition [9].

### Netherlands

The Netherlands initiated a national genome project, Genome of the Netherlands (GoNL; http://www.nlgenome.nl/), in 2009. GoNL is part of a project supported by the Netherland Biobanking and BioMolecular resources Research Infrastructure (BBMRI). The BBMRI has collected biological specimens of more than 600,000 individuals across 180 sample collection sites in the Netherlands, and has generated single nucleotide polymorphism microarray data of 150,000 individuals as of 2014 [2]. GoNL conducted genomic analysis on the inhabitant in 11 areas in the country, and reported the results of WGS analysis performed on 769 individuals. The analysis was based on 231 trios (parents and a single child), 8 families with monozygotic twins, and 11 families with dizygotic twins (Figure 1). Their experimental design allowed improving accuracy and efficiency of haplotyping (Table 2). Historically the Netherlands has suffered damage many times because a large area of the country is below sea level, and because of various sociocultural phenomena that have occurred as a result. Flood control policies—river and water level management—have caused changes in settlement selection by the residents and influenced population migration. Considering such factors, GoNL assumed that historic floods or changes in water level might have impacted on the genetic background and tested it by performing identityby-descent analysis [10]. Furthermore, GoNL provided the frequency of the variants in the Dutch population, used in the WGS analysis of subjected with intellectual disabilities in the Netherlands [11].

### United Kingdom

The UK is currently operating a national genome project called Genomics England (https://www.genomicsengland.co.uk/). In 2012, the UK government established a plan to generate and standardize genomes for clinical use as part of a natural science research scheme of the National Health Service. Genomics England has investigated more than 8,000 rare diseases with a prevalence of less than 0.05% and 7 cancer types commonly reported in the UK population. On research infrastructure and the production of genome data, Genomics England is in partnership with the Wellcome Trust, the Wellcome Trust Sanger Institute, and Illumina, an American company providing sequencing technology. In addition, the Genomics England has collaborated with industry throughout the Genomics Expert Network for Enterprises Consortium, or affiliated with other research institutes for data sharing and participation.

In addition, the UK10K project (http://www.uk10k.org/) started in 2010 in a support of the Wellcome Trust research [12]. It succeeded the 1000 Genome Project with a large number of research participants, and estimate a heritability of quantitative traits from the cohort in UK. The UK10K project began on the basis of two epidemiological cohort studies: Avon Longitudinal Study of Parents and Children [13] and TwinsUK [14]. The project has generated low coverage (7-fold depth) and high coverage (80-fold depth) WES data of 3,781 healthy individuals and analyzed rare variants (allele frequency less than 0.1%) with higher accuracy than the 1000 Genome Project (Figure 1). Furthermore, WGS and WES data allowed genotype imputation of individuals from the epidemiological cohorts [15]. The success of the project was attributed to extensive epidemiological and phenotypic data collected over a long period and studies that have investigated phenotypes corresponding to the genotypes. Moreover, the UK10K project has implemented the analytic framework and resources from the previous genome project, such as the 1000 Genome Project, but also has continued in the Genome England.

### Japan

In 2012, Japan established Tohoku Medical Megabank Organization (ToMMo; http://www.megabank.tohoku.ac.jp/english/), a biological resource organization to collect clinical and genomic resources. They have generated WGS data of 1,070 Japanese from the Tohoku area. With high-coverage WGS data (32-fold depth), they identified rare and copy number variations with low falsediscovery rate, providing the framework for future genomic analysis. In addition, the ToMMo shared data via the Integrative Japanese Genome Variation Database (https://ijgvd.megabank.tohoku.ac.jp/), and has presented resource collation in research field [16].

### Finland

Finland operates a national genome project, the Sequencing Initiative Suomi (SISu), and simultaneously conducts FINRISK (a clinical, epidemiological cohort study) and Health 2000 (an epidemiological study tracking chronic diseases in workers). Recently, SISu researchers revealed low coverage (4.6-fold depth) WGS data of 1,463 Finns in a preprint repository of bioRxiv [3]. This study compared the Finnish data with WGS data of 1,463 Britons provided by UK10K and identified genetic characteristics of the Finns, i.e., several genetic bottlenecks that the Finns experienced throughout the history and the geographical isolation. Furthermore, the distributions of protein-truncating variants found specifically in Finns, and other rare variants were estimated based on the founder effect found in Finns, laying the foundation for future research on rare variant-based disease.

### Sweden

Sweden has SweGen (http://swefreq.nbis.se/). The national genome project is operated by Science for Life Laboratory, a national molecular biology research institution, and funded by a non-profit organization (the Knut and Alice Wallenberg Foundation) and a government research foundation (the National Research Council). In 2011, Sweden analyzed the genotype data of approximately 0.05% of the population and found genetic difference between northern Sweden and the rest of the country [4]. Based on the data, SweGen began to create a reference cohort in the Swedish population. The cohort was recruited at the same time when two epidemiological cohorts, the Swedish Twin Registry [17] and the Northern Sweden Population Health Study [18], were established, and a sample consisting of a total of 1,000 individuals was recruited. SweGen generated high coverage (20-fold depth) WGS data and made public the allele frequency of each variant on SweFreq (https://swefreq.nbis.se) (Figure 1).

## SUGGESTIONS FOR A SUCCESSFUL NATIONAL GENOME PROJECT

### Increasing the level of genome analysis and reproducibility through collaboration and data sharing among consortiums

Researchers with diverse backgrounds have participated in population genetic and genome research, from cohort recruitment to the generation and analysis of genome data. The researchers involved in these areas of research and the data are shared across many consortiums and projects. Novel hypotheses and scientific questions are formulated in the process, and experiments are conducted. Most genome projects operated in several countries are performed in this cultural background. The Netherlands’ GoNL sought to improve the level of analysis and to diversify by including outstanding genome researchers, not only of their own country but also of other countries. To conduct projects, Sweden and Finland recruit the researchers from the UK, the country that initiated a genome project before them, to share their analytical techniques.

Sharing genome data and research methods with a multi-country genome consortium increases the level of genome analysis and reproducibility. As an example, the multi-country genome consortium for WES, the Exome Aggregation Consortium (ExAC), aggregated the raw data generated in medium-scale and smallscale WES studies conducted in various countries, developed a method of genome analysis that could be standardized and reproduced, and reported it with the data to the public [19]. Such efforts of ExAC helped WES data to be utilized in clinical practice and preventive medicine.

### Need for a standardization of genome analysis

A few national genome projects examined competitiveness and conducted quality assurance, before genome data were utilized in practice or research. The US National Institute of Standards and Technology (NIST) attempts to assure the minimum quality in the analysis of bioinformatics used in genome research, through an industry-university cooperative research consortium called Genome in a Bottle (GIAB; https://www.nist.gov/programs-projects/genome-bottle) [20]. NIST/GIAB provided a guideline to ensure the quality of DNA samples used in genome analysis, and referenced the samples from the HapMap project to compare sample standards [20]. By sharing the raw data, they made it possible for other government agencies, academic institutions, and companies to compare analytical methods without generating data. Similar to the 1000 Genome Project, the Netherlands’ GoNL used all possible analytical methods and algorithms to explore structural variations and compared them appropriately in a research article reporting the genomes of the country [10]. Similarly, in the UK, efforts were made in technical improvements and quality comparisons in large-scale WES and WGS research projects (such as UK­ 10K, Developmental Delay Disorders Consortium, Genomics England, etc.).

The need to standardize genome analysis is not simply for accuracy of methods to analyze genomes. With standardization, national-level consortiums can consistently manage data, future associations among consortiums can be enhanced, and the cost for data generation and regeneration/analysis can be managed efficiently (Figure 1). Let us assume the following hypothetical scenario. In 2012, Institution A generated WGS data of 100 individuals in a particular cohort. In the data generated by Illumina, individual genotyping was used with the use of Burrows-Wheeler Aligner (BWA) and Genome Analysis ToolKit unified genotypers. Later in 2016, Institution B generated WGS data of 50 individuals using the Illumina genome platform. The institution used the BWA-Mem, GATK haplotype caller, and multi-sample joint genotyping. Institution C plans to conduct a project on rare variants in Koreans by aggregating two sets of data. In the process, the following points should be considered. What is the margin of error in the accuracy of rare variant calling that occurs because of two different data formats? Is a significant result of the burden or association test really a true positive or a bias occurring because of the data format or difference in quality?

## CONCLUSION

In this report, human genome projects currently underway at the national level were summarized. We live in a time when genome technologies are being actively used in biological and medical research, as well as in clinical practice. At the same time, however, as the press and the public paid great attention, overly optimistic views to rely only on technology have appeared. However, scientific research including biomedicine has developed through the interaction between hypothesis and data. It is to be emphasized that genome research, too, should be used as a resource for not only clinical practice or disease research but also population genetics, human genetics, genomics, and bioinformatics.

Conducting a national genome project requires tremendous amounts of research funding, infrastructure, and human resources. For a national genome project to be successful, the research direction corresponding to the country’s situation and a plan as to how to utilize the results should be clearly established, as seen in several cases discussed above. The plan on how to progress with the project should be published in an scientific journal, as the Netherlands and Finland have done, and a platform should be created for discussion among international scientific organizations. Through this process, researchers can address in the national genome project what are currently utilized or discussed in the scientific field, while the government sets the stage for the researchers to be in a leading position to conduct the project.

## Figures and Tables

**Table 1.** Types and characteristics of mutations reported in genetic disorder research

Variant type	Allele frequency (%)	Genetic data format	No. per genome
Common	≥5.0	GWAS; resequencing; WES; WGS	3-4 million
Low frequency	0.1-5.0	GWAS; resequencing; exome array; WES; WGS	Various by ancestry background
Private	<0.1	WES; WGS	Differ by individual

GWAS, genome-wide association study; WES, whole exome sequencing; WGS, whole genome sequencing.

**Table 2.** Analytic details of the national genome projects

	Sequencing source	Reads mapping	Variant calling	Haplotyping	Genotype imputation
Sweden	Internal	BWA-Mem	GATK-HC	In progress	In progress
Finland	External	BWA	GATK-UG	Completed	Completed
United Kingdom	Internal + external	BWA	Samtools; bcftools	Completed	Completed
Iceland	Internal	BWA	GATK-UG	Completed	Completed
Japan	Internal	BWA-Mem	GATK-UG; bcftools	Completed	Completed
Netherlands	External	BWA	GATK-UG	Completed	Completed

BWA, Burrows-Wheeler Aligner; GATK, Genome Analysis Toolkit; HC, haplotypecaller; UG, unified genotyper.
