# Supplementary material for: National human genome projects: an update and an agenda
Source: Epidemiol Health. 2017 Oct 16;39:e2017045. doi: 10.4178/epih.e2017045 (PMC5675980; doi:10.4178/epih.e2017045)
Supplement: Supplementary file 1 [file epih-39-e2017045-supplementary.pdf]

Volume: 39, Article ID: e2017045
https://doi.org/10.4178/epih.e2017045

National Human Genome Projects: An Update and an Agenda

Joon Yong An

Department of Psychiatry, Weill Institute for Neurosciences, University of California, San Francisco, CA, USA

Correspondence: Joon Yong An Department of Psychiatry, Weill Institute for Neurosciences, University of California, 4th Street, San Francisco, CA 94158, USA E-mail: joonyong.an@ucsf.edu

Volume: 39, Article ID: e2017045

https://doi.org/10.4178/epih.e2017045

ABSTRACT

Population genetic and human genetic studies are being accelerated with genome technology and data sharing. Accordingly, in the past 10 years, several countries have initiated genetic research using genome technology and identified the genetic architecture of the ethnic groups living in the corresponding country or suggested the genetic foundation of a social phenomenon. Genetic research has been conducted from epidemiological studies that previously described the health or disease conditions in defined population. This perspective summarizes national genome projects conducted in the past 10 years and introduces case studies to utilize genomic data in genetic research.

KEY WORDS: Genomics, Human genome project, Next generation sequencing, Whole genome sequencing, Genetics, Microarray

서론

지난 한세기 동안, 유전학(genetics)은 인류 사회에서 일어나는 현상들을 유전적인 요인들을 이용하여 설명해왔다. 특히, 유전체 해독 기술(genome sequencing technology) – Microarray 와 next generation sequencing 의 발달에 따라, 지난 10 년동안 인간 질병 (disorders) 및 형질(traits)에 관한 유전적인 원인들을 밝히는 연구가 활발히 이뤄졌다. 인간 유전학에 대한 공동의 노력인 다국적 유전체 연구 컨소시엄이 시작되었다. 1000 Genome Project 은 5 종류의 인구집단(population)과 26 종류의 세부 인구집단에 대한 유전체 정보를 제공한다(1). 이 컨소시엄은 2009 년부터 2015 년까지, 각 인구 집단 별 low coverage (2-4X depth) WGS (whole genome sequencing) 데이터를 단계적으로 공개하기 시작했다. 이를 통해, 연구자들은 인구 집단의

혈통(population ancestry)마다 특이적으로 발견되는 변이(예. fixed allele)나 집단 내의 1% 미만의 빈도수로 나타나는 rare variant 의 분포를 보고했다. 또한 1000 Genome Project 내의 WGS 데이터는 유전적으로 유사한 인구 집단 별 유전형을 추정하는 imputation 의 참조 자료로 활용되고 있다. 현재, 여러 국가들은 각 국가를 거주하는 인구 집단에 대한 유전적인 조성에 대한 유전체 프로젝트를 진행하고 있고, WGS 분석에 힘쓰고 있다.

국가 유전체 연구는 사회 구성원을 유전적으로 묘사할 수 있는 기회를 제공한다. 형질 (trait) 혹은 질병에 대한 연구는 국가를 구성하는 사회 구성원들의 유전적인 분포도를 이해하는 수단으로 사용된다. 따라서, 유전체 데이터는 국가는 사회구성원들의 건강과 복지를 위한 유용한 자원으로 활용될 수 있다. 특히, 형질의 분포도에서 극단치(outlier)에 해당하는 구성원들 - 희귀 질환 환자 등등 -은 일차적인 혜택을 받게 된다. 따라서, 국가 유전체 프로젝트는 한 국가를 구성하는 인구 집단을 기술하면서 동시에, 현재 국가가 당면하고 있는 사회적인 현상을 이해하는 도구가 된다.
이 persepctive 에서는 현재 진행 중인 국가별 유전체 프로젝트에 대하여 소개한다. 현재 차세대 유전체 분석 방법(whole exome sequencing 및 whole genome sequencing)을 이용하여 진행 중인 유전체 프로젝트를 정리하고, 각 프로젝트의 당위성과 데이터 수집 및 활용 방안을 정리한다. 또한, 각 프로젝트의 자료를 이용한 유전학 연구들의 사례들을 소개한다.

국가 유전체 프로젝트의 당위성

국가 거주민의 유전적 배경

유전체 연구는 한 영토에 거주하는 주민들의 유전적인 배경을 살펴보거나, 국가 내 인구집단(population)에서 일어난 사회 현상에 대한 결과를 유전적 조성을 토대로 파악한다. 아이슬란드, 네덜란드를 비롯한 유럽 국가들은 유전체 정보를 통해, 각 국가에 장기간 거주한 인구 집단을 연구하였다. 아이슬란드는 북유럽에서 오랜동안 기록된 가족력과 지리적으로 고립된 인구 집단의 특성을 이용하여, 인간 유전학 및 집단 유전학에 관련된 기초적인 질문들을 연구하였다. 한편, 네덜란드는 도시국가 형태를 유지해온 역사를 근거로 하여, 11 개 지역을 세부 인구 집단들로 분류하고, 국가 유전체 프로젝트에 참여시켰다(2). 핀란드와 스웨덴의 국가 유전체 프로젝트는 영토 내 이주 및 fitness 에 영향을 미칠 수 있는 몇몇 역사적인 사건을 바탕으로 각 국민의 이질적인 유전적인 조성을 밝혀냈다(3,4). 영국은 다인종 경시적 코호트 연구인 Born in Bradford 을 통해, 영국 Bradford 도시에 거주하는 파키스탄계 영국인을 주목했고, 이들의 유전적 거리가 가까울 것이라는 가설을 수립하였다. 코호트에 포함된 3,222 명의 WES 을 분석함으로써, homozygous variant 가 실제 형질이나 질병과 연관이 있는지에 대해 조사하였다(5).

질병 연구를 위한 유전체 연구

국가 유전체 프로젝트는 어떤 인구 집단에게 흔하게 발견되는 질환에 대한 보건, 유전학적 지표를 마련하고, 질병 위험요인을 규명하는 것을 목표로 한다. 인류 집단에서 매우 낮은 빈도수로 나타나는 변이들은 인구집단 특이적(population

specific)이며, 해당 인구 집단이나 지역거주민에게 특징적으로 발견되는 질병의 원인이 될 수 있다 (6). 그러나, 특정 인구 집단의 유전체 데이터를 연구하는 것이 특정 질병을 연구하는것에 타당한지를 고려해야한다. 질병과 연관된 변이(associated variant)나 질병을 일으키는 변이(causal variant)들은 여러가지 종류가 있다. GWAS 를 이용한 common variant 탐색과 가계도 WES 분석을 이용한 de novo variant 는 현재까지 가장 잘 연구된 변이들이며, 대규모 코호트 연구에서 유전학적 구성 (genetic architecture) 및 effect size 가 잘 알려진 변이들이다. 인구 집단에 대하여 특이적인 rare variant 는 특정 인구 집단 집단에서 흔하게 발견되는 질병이나 형질에 대한 유전적 요인을 설명해줄 것으로 기대한다. 하지만, rare variant 의 effect size 를 정교하게 측정하기 위해서는 많은 수의 표본이 요구된다(7).

따라서, 국가가 질병연구와 임상 유전체를 위해 유전체 프로젝트를 시작해야한다는 명분은 국가 영토에 거주해온 인구의 유전적 특성을 고려하는 것, 역학 코호트 연구와의 연계, 혹은 유전적인 가설이 충분히 뒷받침 되어야 한다. 예를 들어, 아이슬란드는 국가 유전체 프로젝트에서 그들의 인구 집단이 유전적으로 균일하다는 점을 고려하여, 건강과 질병에 큰 영향을 미치는 protein-truncating variant 가 실제 국민의 건강에 영향을 미치는지를 조사하였다(8). 이 연구에서 연구자들은 2,600 여명의 WGS 데이터를 생산했고, 이를 이용해 기존에 수집한 십만명의 유전형 데이터를 imputation 을 했다. 그리고 풍부한 가족력 정보와 표현형 데이터, 사망률을 집계하여 실제 이러한 종류의 변이가 질병에 영향을 미치는지, 얼마만큼 낮은 빈도수가 변이의 penetrance 에 기여하는지, homozygous variant - 지리적인 근접도와 유전적인 근접도로 치환되는 변이의 종류- 가 실제 질병에 대해 높은 effect

size 를 갖는지에 대하여 조사하였다. 이 연구의 성공 요인은 단순히 양질의 최신 WGS 데이터를 사용했던 것에 기인하지 않는다. 이들은 기존 유전학 연구(유전형 수집 및 유전적 근사도 추정)을 적극적으로 활용한 것, 아이슬란드 상황에 부합하는 유전적인 가설과 적합한 실험 설계가 이뤄졌다는 점에 있다.

Volume: 39, Article ID: e2017045

https://doi.org/10.4178/epih.e2017045

표 1. 질병 유전학 연구에서 보고되는 변이 종류와 특성

Type	Allele frequency	Genetic data format	Number per genome
Common variant	≥5%	GWAS; resequencing; WES; WGS	3-4 million
Low frequency variant	0.1-5%	GWAS; resequencing; exome array; WES; WGS	Various by ancestry background
Private variant	<0.1%	WES; WGS	Differ by individual

국가별 유전체 프로젝트 사례

아이슬란드

아이슬란드는 현재 국가 수준의 유전체 연구를 선도하는 국가이며, 또한 현대 인간 유전학의 많은 기여를 해온 국가이다. 인간게놈프로젝트의 초안이 발표되기 전, 1996 년 무렵, deCODE Genetics (https://www.decode.com)라는 아이슬란드의 벤쳐 회사는 전 국민을 상대로한 유전체 분석을 하겠다는 계획을 발표했다. 이 계획이 타당했던 이유는, 아이슬란드는 9 세기 북유럽에서 2 만명 가량이 이주하여 시작된 국가이면서, 지리적인 고립으로 인한 genetic admixture 가 적은 small founder 의 유전적 모델로 적합한 인구 집단이었기 때문이다. 게다가, 아이슬란드는 이주 이후로 가족에 대한 정보를 기록하고 후세에게 물려주는등 가족력에 대한 풍부한 정보를 보유하고 있다. deCODE Genetics 는 아이슬란드의 풍성한 가족력 정보와 유전적으로 균일한 인구집단이라는 이점을 이용해, 유전체 연구를 하는 것이 질병이나 형질에 대한 유전적 기여도를 찾는데 충분하다고 판단했다. 유전체 데이터를 생산 한 이후 (figure 1), 이들은 집단 유전학에서 갖는 여러가지 이론들과 가설들 – paternal age in de novo variant, gene conversion error, and recessive model in health condition – 을 실험하였다 (9).

네덜란드

네덜란드 2009 년 국가 유전체 프로젝트 Genome of the Netherlands (GoNL; http://www.nlgenome.nl/)를 시작하였다. GoNL 은 the Netherland

Volume: 39, Article ID: e2017045
https://doi.org/10.4178/epih.e2017045

Biobanking and BioModelcular resources Research Infrastructure (BBMRI)의 지원 사업 의 일부이다. BBMRI 는 네덜란드 내의 180 군데의 샘플 수집처에서 60 만명 이상의 생물학 시료를 수집하였고, 10-15 만명의 SNP microarray 데이터를 생산중이다 (2014 년 기준)(2). GoNL 은 네덜란드 11 개 지역의 원주민들에 대한 유전체 분석을 시행하였고, 2014 년 769 명의 WGS 분석을 공개하였다. 이는 231 삼인 가족 (부모 – 자녀; trio)와 일난성 쌍둥이 8 가족, 이란성 쌍둥이 11 가족을 포함한다(figure 1). 이러한 실험 설계를 통해, haplotyping 의 정확성과 효용성을 높였다. 네덜란드는 해수면 보다 낮은 국토 때문에 역사적으로 많은 피해를 입었고 이에 따른 여러 사회문화 현상들이 발생했다. 치수정책-하천 및 해수면 관리-가 국민들의 거주지 선택에 변화를 주거나 , 인구 이동에 영향을 미쳤다. GoNL 은 이점을 토대로, 역사적인 홍수나 해수면 변화에 따른 유전적 조성의 변화가 있을것이라는 가설을 수립했고, identity-by-descent 분석을 이용하여 살펴보았다(10). 이 뿐만 아니라, GoNL 는 네덜란드 인구 집단에 대한 보다 정확한 변이의 빈도수를 제공함으로써, 네덜란드 내의 지적장애인 50 명에 대한 WGS 분석에 활용되었다(11).

영국

영국은 Genomics England (https://www.genomicsengland.co.uk)라는 국가 유전체 프로젝트를 운영하고 있다. 2012 년 영국 정부는 The National Health Service (NHS)의 자연과학 연구계획의 일환으로 임상 유전체 생산 및 표준화를 계획한다. Genomics England 는 영국인 인구 중, 0.05% 미만의 유병률을 갖는 8,000 개 이상의 희귀 질환 혹은 영국인에게 많이 발견되는 7 개의 암 종류들을 연구한다. 연구 인프라와 유전체 데이터 생산은 Wellcome Trust, the Wellcome Trust Sanger Institute (WTSI) 그리고 미국 유전체 회사 Illumina 와

파트너쉽을 맺었다. 이와 더불어, 유전체 데이터 활용 및 참여에 대해서는 Genomics Expert Network for Enterprises 컨소시엄을 통해 산업체들과 협력하거나, 타국의 유전체 연구 기관과 제휴를 맺었다.

이외에도 영국은 2010 년 Wellcome Trust 의 연구 지원을 통해 UK10K 프로젝트(www.uk10k.org)를 진행하고 있다(12). 이는 영국의 연구진들이 1000 Genome Project 에 대거 참여한 이후 계획되었다. 이를 통해, 영국인에게 특이적으로 발견되는 양적형질에 초점을 맞추었고, 이에 대한 유전력 및 유전적인 기여도를 찾기 위해 이 프로젝트를 시작했다. UK10K 는 두개의 역학 코호트들 - Avon Longitudinal Study of Parents and Children(13)과 TwinsUK(14) -을 바탕으로 시작하였다. 이 프로젝트는 healthy individual 을 3,781 명에 대한 low coverage WGS 데이터 (~7x)와 high coverage WES 데이터 (~80x)를 생산하여, 1000 Genome Project 보다 더 정확한 방식으로 rare variant(allele frequency 0.1% 미만)을 분석했다(figure 1). 또한 WGS, WES 데이터를 이용해, 역학 코호트에서 생산된 genotyping 데이터에 대해 보다 정교한 imputation 이 가능하도록 하였다(15). 국가 유전체 프로젝트로서, UK10K 가 성공적인 이유는 오랜 기간동안 수집된 광범위한 역학 데이터 및 표현형 데이터를 수집했다는 점과 이를 토대로 유전형에 부합하는 표현형을 분석하려 했다는 점이다. 또한 1000 Genome Project 와 같은 기존의 유전체 프로젝트의 전승을 따르고, Genomics England 와 같은 후속 프로젝트에 분석방법과 인적자원을 계승하였다.

Volume: 39, Article ID: e2017045
https://doi.org/10.4178/epih.e2017045

일본

일본은 2012 년 도호쿠 지방에 Tohoku Medical Megabank Organization (ToMMo; http://www.megabank.tohoku.ac.jp/english/)를 설립함으로써, 의학과 유전체 정보를 수집하는 생물학 자원 센터를 설립했다. 2014 년, 토호쿠 지역 일본인 1,070 명의 WGS 데이터를 생산하고 학계에 진행 상황을 보고하였다. 이 프로젝트에서 high-coverage 데이터(~32X)를 이용해, 낮은 위양성률(false-discovery rate)로 인구 집단의 특이적인 rare variant 와 복제수 변이를 찾는 등, 향후 유전체 분석을 위한 기초를 마련했다 . 이와 더불어, 일본은 ToMMo 의 데이터를 the integrative Japanese Genome Variation Database(https://ijgvd.megabank.tohoku.ac.jp/)를 통해 공유하고, 수집된 정보를 학계에 보고하였다 (16).

핀란드

핀란드는 Sequencing Initiative SUomi (SISU)라는 국가 유전체 프로젝트를 운영하며, FINRISK 라는 임상, 역학 코호트 연구 그리고 근로자들의 만성질환을 추적하는 Health 2000 역학 연구와 함께 진행되고 있다. 최근 bioRxiv 에 공개된 preprint 에서, SISU 는 1,463 명의 핀란드인의 low coverage (~4.6X)의 WGS 데이터를 공개하였다(3). 이 연구는 영국의 UK10K 에서 제공된 1,463 명의 영국인 WGS 데이터와 비교하였고, 핀란드인이 역사적으로 겪은 여러번의 genetic bottleneck 과 지리적으로 고립되었다는 유전적인 특성을 찾게 되었다. 또한 핀란드인에게 나타나는 founder effect 을 바탕으로, 핀란드인들에게 특이적으로 발견되는 protein-truncating 변이 및 기타 rare variant 의 분포도를 산출하였고, 향후 rare variant 기반 질환 연구에 대한 기초를 마련하였다.

Volume: 39, Article ID: e2017045
https://doi.org/10.4178/epih.e2017045

스웨덴

스웨덴은 국가 유전체 프로젝트 SweGen(swefreq.nbis.se)를 운영하고 있다. 이는 분자생물학 국가 연구기관인 Science for Life Laboratory 에 의해 진행되며, 비영리 재단인 the Knut and Alice Wallenberg Foundation 과 정부 연구재단의 The National Research Council 지원으로 이뤄진다. 스웨덴은 2011 년 인구의 0.05% 가량의 유전형 정보를 수집하였고, 스웨덴 북부와 나머지 지역의 유전적 차이가 있음을 밝혔다(4). 이러한 데이터를 기반으로, SweGen 은 스웨덴 인구의 참조 코호트 작성을 시작했다. 코호트 수집은 두가지 역학 코호트들 - the Swedish Twin Registry(17)와 the Northern Sweden Population Health Study(18) -과 함께 이뤄졌으며, 이를 통해 1,000 명의 표본을 모집했다. SweGen 은 high coverage WGS 데이터(20x)를 생산했고, SweFreq(https://swefreq.nbis.se)를 통해 각 변이들의 allele frequency 를 공개했다 (figure 1).

성공적인 국가 유전체 프로젝트를 위한 제안

컨소시엄 간 협력과 공유를 통한 분석 수준 향상과 재현성 확보

집단유전학과 유전체 연구는 코호트 수집부터, 유전체 데이터 생산, 분석에 이르기까지 여러 연구 배경을 가진 인적 자원들이 참여해왔다. 이에 참여한 사람들과 데이터들이 여러 컨소시엄과 프로젝트에 공유되고, 이동하면서 새로운 가설과 과학적인 질문들을 시도하고, 실험하게 된다. 현재 운영되는 여러 나라들의 유전체 프로젝트 대부분이 이러한 문화를 바탕으로 이뤄지고 있다. 네덜란드의 GoNL 의 경우, 자국 이외의 해외 유수 유전체 인력을 프로젝트에

포함시킴으로써 분석 수준 향상 및 다변화를 모색했다. 스웨덴과 핀란드는 유전체 프로젝트를 먼저 시행했던 영국의 연구자들을 자국의 프로젝트에 참여시키고, 분석 방법을 공유한다.

유전체 데이터 및 연구 방법을 다국적 유전체 컨소시엄과 공유하는 것은 유전체 분석의 수준의 향상 시키고 연구의 재현성을 확보한다. 가령, WES 의 다국적 컨소시엄 The Exome Aggregation Consortium (ExAC)은 다양한 나라의 중,소규모의 WES 연구들에서 생산된 raw 데이터를 취합했다. 이들은 수년동안, 표준화되고 재현 가능한 유전체 분석방법을 개발하고, 이를 데이터와 함께 일반에게 공개하였다(19). ExAC 의 노력으로 인해, 임상 및 예방 의학에서 WES 결과를 활용하는데 도움을 주었다.

유전체 분석 표준의 필요성

몇몇 국가 유전체 프로젝트들은 유전체 데이터가 임상이나 연구에 사용되기 전, 충분한 경쟁력과 품질을 갖도록 살펴보았다. 미국 국립 표준기술 연구소(National Institute of Standards and Technology; 이하 NIST)는 Genome in a Bottle (https://www.nist.gov/programs-projects/genome-bottle)이라 불리는 산학연계 컨소시엄을 통해, 유전체 연구에서 사용되어야 하는 최소한의 품질을 확보하도록 노력했다(20). NIST 는 HapMap 프로젝트 샘플 혹은 3 인 가족들을 여러 유전체 해독 플랫폼에서 데이터를 생산했고, raw 데이터를 공개하였다. 이로인해, 타 국가기관이나 학계, 회사들이 데이터 생산 없이, 분석방법들을 비교할 수 있는 자리를 마련했다. 네덜란드의 GoNL 은 국가 유전체를 보고하는 연구 논문에서, 구조 변이를 탐색하기 위해, 1000 Genome Project 와 마찬가지로 가능한 모든 분석 방법과 알고리즘을 사용했고, 이를

Volume: 39, Article ID: e2017045
https://doi.org/10.4178/epih.e2017045
적절하게 비교하였다(10). 마찬가지로, 영국의 경우, UK10K, Developmental Delay Disorders Consortium, Genomics England 등 대규모 WES 및 WGS 연구에서 기술적인 보완과 품질 비교를 하려고 노력했다.

유전체 분석 표준의 필요성은 단순히 유전체 분석기술의 정확도만을 위함이 아니다. 국가 단위 컨소시엄의 지속적인 데이터 관리와 차후 컨소시엄간의 연계성을 높이고, 데이터 생산과 재생산/분석에 들어가는 비용을 효율적으로 관리할 수 있다 (figure 1). 가령 다음과 같은 상황을 가정해보자. 2012 년에 기관 A 는 특정 코호트 100 명의 WGS 데이터을 생산했다. Illumina 에서 얻어진 데이터는 BWA 와 GATK Unified genotyper 에 의해 individual genotyping 방식을 이용하였다. 이후 2016 년, 기관 B 는 50 명의 WGS 데이터를 Illumina 유전체 플랫폼을 이용해 생산했다. 기관 B 는 BWA-Mem, GATK Haplotype caller 와 multi-sample joint genotyping 을 이용하였다. 기관 C 는 이 두가지 데이터를 수합하여, 한국인에게 rare variant 에 관한 프로젝트를 진행하려고 한다. 이 과정에서 고려해야 할 사항은 다음과 같다. Rare variant calling 시, 데이터의 두가지 형식에 의해 발생할 수 있는 정확도의 오차 범위는 얼마만큼이 될까? Burden 및 association test 의 유의한 결과가 실제 true positive 인가, 아니면 데이터의 형식 및 품질의 차이로 발생하는 bias 인가?

결론

이 보고서는 현재 국가 단위로 진행 중인 인간 유전체 프로젝트에 대하여 정리하였다. 지금은 유전체 기술이 생물학, 의학 연구 및 임상에 활발히 이용되는

Volume: 39, Article ID: e2017045
https://doi.org/10.4178/epih.e2017045
시대이다. 하지만 동시에 언론과 대중에 많은 주목을 받으면서, 기술에만 의존하는 지나친 낙관론이 등장하기도 한다. 하지만, 생의학을 포함한 과학 연구는 가설과 데이터의 상호작용으로 발전해왔으며, 유전체 연구도 임상 및 질병연구 뿐 아니라 집단 유전학, 인간 유전학, 유전체학, 생명정보학을 위한 자원으로 활용되어야 함을 강조한다.

국가 유전체 프로젝트는 막대한 연구비, 인프라 그리고 인적 자원을 필요로 한다. 몇몇 국가의 사례에서 보았듯이, 성공적인 프로젝트를 위해, 각 국가에 부합하는 연구 방향성과 활용방안에 대해 분명한 계획을 제시해야한다. 네덜란드 및 핀란드와 같이 프로젝트의 진행계획에 대해 학술지에 게재해야하며, 국제 학계와 논의할 수 있는 통로를 만들어야 한다. 이런 과정을 통해, 연구자들은 학계에서 활용되거나 논의되는 사안에 대하여, 프로젝트에 적용할 수 있으며, 정부는 연구자들이 프로젝트를 주도적으로 진행할 수 있는 자리를 마련해준다.

참고 문헌

1. 1000 Genomes Project Consortium, Abecasis GR, Altshuler D, Auton A, Brooks LD, Durbin RM, et al. A map of human genome variation from population-scale sequencing. Nature 2010;467:1061-1073
2. Boomsma DI, Wijmenga C, Slagboom EP, Swertz MA, Karssen LC, Abdellaoui A, et al. The Genome of the Netherlands: design, and project goals. Eur J Hum Genet 2014;22:221-227.
3. Chheda H, Palta P, Pirinen M, McCarthy S, Walter K, Koskinen S, et al. Whole-genome view of the consequences of a population bottleneck

using 2926 genome sequences from Finland and United Kingdom. Eur J Hum Genet 2017;25:477-484.
4. Humphreys K, Grankvist A, Leu M, Hall P, Liu J, Ripatti S, et al. The genetic structure of the Swedish population. PLoS One 2011; 6:e22547.
5. Narasimhan VM, Hunt KA, Mason D, Baker CL, Karczewski KJ, Barnes MR, et al. Health and population effects of rare gene knockouts in adult humans with related parents. Science 2016;352:474477.
6. Sanna S, Li B, Mulas A, Sidore C, Kang HM, Jackson AU, et al. Fine mapping of five loci associated with low-density lipoprotein cholesterol detects variants that double the explained heritability. PLoS Genet 2011;7:e1002198.
7. Lee S, Abecasis GR, Boehnke M, Lin X. Rare-variant association analysis: study designs and statistical tests. Am J Hum Genet 2014; 95:5-23.
8. Sulem P, Helgason H, Oddson A, Stefansson H, Gudjonsson SA, Zink F, et al. Identification of a large set of rare complete human knockouts. Nat Genet 2015;47:448-452.
9. Letters from Iceland. Nat Genet 2015;47:425.
10. Genome of the Netherlands Consortium. Whole-genome sequence variation, population structure and demographic history of the Dutch population. Nat Genet 2014;46:818-825.
11. Gilissen C, Hehir-Kwa JY, Thung DT, van de Vorst M, van Bon BW, Willemsen MH, et al. Genome sequencing identifies major causes of severe intellectual disability. Nature 2014;511:344-347.

Volume: 39, Article ID: e2017045
https://doi.org/10.4178/epih.e2017045

12. UK10K Consortium, Walter K, Min JL, Huang J, Crooks L, Memari Y, et al. The UK10K project identifies rare variants in health and disease. Nature 2015;526:82-90.

13. Boyd A, Golding J, Macleod J, Lawlor DA, Fraser A, Henderson J, et al. Cohort profile: the 'children of the 90s'--the index offspring of the Avon Longitudinal Study of Parents and Children. Int J Epidemiol 2013;42:111-127.

14. Moayyeri A, Hammond CJ, Hart DJ, Spector TD. The UK Adult Twin Registry (TwinsUK Resource). Twin Res Hum Genet 2013; 16:144-149.

15. Huang J, Howie B, McCarthy S, Memari Y, Walter K, Min JL, et al. Improved imputation of low-frequency and rare variants using the UK10K haplotype reference panel. Nat Commun 2015; 6:8111.

16. Nagasaki M, Yasuda J, Katsuoka F, Nariai N, Kojima K, Kawai Y, et al. Rare variant discovery by deep whole-genome sequencing of 1,070 Japanese individuals. Nat Commun 2015;6:8018.

17. Magnusson PK, Almqvist C, Rahman I, Ganna A, Viktorin A, Walum H, et al. The Swedish Twin Registry: establishment of a biobank and other recent developments. Twin Res Hum Genet 2013;16:317-329.

18. Johansson A, Marroni F, Hayward C, Franklin CS, Kirichenko AV, Jonasson I, et al. Common variants in the JAZF1 gene associated with height identified by linkage and genome-wide association analysis. Hum Mol Genet 2009;18:373-380.

Volume: 39, Article ID: e2017045
https://doi.org/10.4178/epih.e2017045

19. Lek M, Karczewski KJ, Minikel EV, Samocha KE, Banks E, Fennell T, et al. Analysis of protein-coding genetic variation in 60,706 humans. Nature 2016;536:285-291.

20. Zook JM, Chapman B, Wang J, Mittelman D, Hofmann O, Hide W, et al. Integrating human sequence data sets provides a resource of benchmark SNP and indel genotype calls. Nat Biotechnol 2014; 32:246-251.
